# Intranasal administration of α-synuclein preformed fibrils triggers microglial iron deposition in the substantia nigra of *Macaca fascicularis*

**DOI:** 10.1038/s41419-020-03369-x

**Published:** 2021-01-13

**Authors:** Jian-Jun Guo, Feng Yue, Dong-Yan Song, Luc Bousset, Xin Liang, Jing Tang, Lin Yuan, Wen Li, Ronald Melki, Yong Tang, Piu Chan, Chuang Guo, Jia-Yi Li

**Affiliations:** 1 Institute of Neuroscience, College of Life and Health Sciences, Northeastern University, Shenyang, 110819 China; 2 Beijing Key Laboratory of Parkinson’s Disease, National Clinical Research Center for Geriatric Disorders, Department of Neurobiology, Xuanwu Hospital of Capital Medical University, Beijing, 100053 China; 3 Laboratory of Neurodegenerative Diseases, CNRS and Institut François Jacob (MIRCen), CEA, Fontenay-aux-Roses, 92260 France; 4 Department of Histology, Chongqing Medical University, Chongqing, 400000 China; 5 Unit of Neurodegenerative Diseases and Repair, Institute of Health Sciences, China Medical University, Shenyang, 110112 China; 6 Neural Plasticity and Repair Unit, Wallenberg Neuroscience Center, Lund University, Lund, 22184 Sweden

**Keywords:** Parkinson's disease, Parkinson's disease

## Abstract

Iron deposition is present in main lesion areas in the brains of patients with Parkinson’s disease (PD) and an abnormal iron content may be associated with dopaminergic neuronal cytotoxicity and degeneration in the substantia nigra of the midbrain. However, the cause of iron deposition and its role in the pathological process of PD are unclear. In the present study, we investigated the effects of the nasal mucosal delivery of synthetic human α-synuclein (α-syn) preformed fibrils (PFFs) on the pathogenesis of PD in *Macaca fascicularis*. We detected that iron deposition was clearly increased in a time-dependent manner from 1 to 17 months in the substantia nigra and globus pallidus, highly contrasting to other brain regions after treatments with α-syn PFFs. At the cellular level, the iron deposits were specifically localized in microglia but not in dopaminergic neurons, nor in other types of glial cells in the substantia nigra, whereas the expression of transferrin (TF), TF receptor 1 (TFR1), TF receptor 2 (TFR2), and ferroportin (FPn) was increased in dopaminergic neurons. Furthermore, no clear dopaminergic neuron loss was observed in the substantia nigra, but with decreased immunoreactivity of tyrosine hydroxylase (TH) and appearance of axonal swelling in the putamen. The brain region-enriched and cell-type-dependent iron localizations indicate that the intranasal α-syn PFFs treatment-induced iron depositions in microglia in the substantia nigra may appear as an early cellular response that may initiate neuroinflammation in the dopaminergic system before cell death occurs. Our data suggest that the inhibition of iron deposition may be a potential approach for the early prevention and treatment of PD.

## Introduction

Parkinson’s disease (PD) is a common neurodegenerative disease. The motor symptoms of PD include resting tremor, bradykinesia, rigidity, and postural abnormalities^1^. PD patients can also exhibit different nonmotor symptoms, such as constipation^2^, depression^3^, and cognitive decline^4^, which usually precede the motor symptoms. Clinically, there are no effective early diagnostic or therapeutic tools for PD. Thus, robust biomarkers for the identification of PD are essential for early diagnosis and interventions^5^.

The main pathological features of PD include the degeneration of dopaminergic neurons in the substantia nigra and the appearance of Lewy bodies, Luria-Bertani medium (LBs) in surviving neurons^6^. The protein α-synuclein (α-syn) is a constitutive component of LBs that is normally a monomeric protein under physiological conditions but forms amyloid fibrils under pathological conditions. The aggregation of α-syn is closely associated with PD progression. Emerging evidence has shown that the pathological propagation of α-syn in the brain may contribute to PD progression^7^. Multiple risk factors have been shown to jointly contribute to the development of PD, including genetic and environmental factors^8–10^.

Iron is among the most essential trace elements in the human body. Excessive amounts of iron have toxic effects on the nervous system. The factors associated with an increased total iron concentration mainly include aging, the inflammatory response, changes in iron balance, redistribution of iron in the brain, and increased blood–brain barrier permeability^11,12^. Many studies have demonstrated that the iron content in the substantia nigra of PD patients is significantly increased, accompanied by changes in iron metabolism. Therefore, it has been proposed that iron may cause neuronal death by promoting the production of hydroxyl radicals^13,14^. Both epidemiological investigations and related experimental studies have shown that iron is closely associated with the pathogenesis of PD^15^. The accumulation of iron in neurons may cause apoptotic damage^16^ and the accumulation of iron in glial cells induces an inflammatory state by increasing the release of proinflammatory cytokines, leading to neuroinflammation^17^. Semiquantitative histochemical assays of postmortem PD brains have shown that Fe^3+^ is significantly increased in the substantia nigra pars compacta. Indeed, increased iron deposits in neurons and glia and the proliferation of iron-containing microglia have been reported in PD^18–20^. Moreover, ferric iron has been shown to promote α-syn aggregation in vitro^21,22^. In the in situ detection of redox-active iron, neurons in the substantia nigra pars compacta of patients with PD showed strong LBs labeling, which is consistent with the PD oxidative stress hypothesis^23^. In patient brains with more severe neuronal loss, the content of redox-active iron is even higher^24^. Furthermore, an increase in the iron content in the substantia nigra can be observed even in the early stages of PD^25^, suggesting that iron accumulation can be used as an indicator to detect the pathological processes of PD and provide a new target for PD prevention and treatment.

To date, how different brain regions maintain iron concentrations under normal physiological conditions and how the iron distribution changes with inflammatory damage remain unclear. Whether iron deposition occurring in neurodegenerative diseases is a primary event or a secondary effect remains to be elucidated^26^. Previous studies investigating iron regulation have heavily relied on healthy rodents, but iron regulation in rodents differs from that in humans. Therefore, to study the pathogenesis of PD, we generated a novel animal model in which α-syn preformed fibrils (PFFs) were directly administered on the surface of the olfactory epithelium and additionally injected into the olfactory submucosa of monkeys. Robust iron deposits appeared in the substantia nigra and globus pallidus of the animals exposed to α-syn PFFs. At the cellular level, the iron deposition appeared specifically localized in microglia, rather than in dopaminergic neurons nor other types of glia. Although we did not detect clear dopaminergic neuronal loss in the substantia nigra, we observed signs of neurodegenerative alterations in dopaminergic terminals in the putamen, suggesting that iron deposition in microglia may precede dopaminergic neuronal death.

## Materials and methods

### Monkeys used in the project

In total, 12 male cynomolgus monkeys (*Macaca fascicularis*) ranging in age from 6 to 10 years (similar to human adulthood) were selected and randomly divided into the following two groups: α-syn PFFs groups (*n* = 9) and control group treated with phosphate-buffered saline (PBS) (*n* = 3). The monkeys were maintained at the Grandforest Primate Breeding Co. Ltd, Guangxi, China, and all experimental animals had detailed birth records and quarantine certificates.

In the monkeys exposed to the α-syn PFFs, 20 μl of 1 μg/μl α-syn PFFs was smeared unilaterally (right side) on the olfactory epithelial mucosa, followed by injection (20 μl) into the olfactory epithelium. The total volume of the two administrations was 40 μl. The treatment was performed once a week for 4 weeks. The animals were killed and brain samples were collected 1, 4, and 17 months after α-syn administration; there were three monkeys in each time point (Supplementary Fig. 1).

Under deep anesthesia with pentobarbital sodium, the heart of each monkey was exposed and intracardially perfused with 2–3 l of 0.01 M PBS, followed by 4% paraformaldehyde (PFA) solution. Some monkey brains were perfused with PBS alone, removed, and cut into 2 or 4 mm-thick brain slices. The 2 mm-thick brain slices were immediately placed on dry ice and stored in a −80 °C freezer until use and the 4 mm-thick brain slices were fixed in 4% PFA for the paraffin-embedding processes. The remaining monkey brains were cut into anterior, middle, and posterior blocks and directly placed in 4% PFA for post-fixation over two nights. The fixed monkey brains were cut into 40 μm-thick sections.

### Generation of α-syn PFFs

The expression and purification of human wild-type (WT) α-syn was performed as previously described^27^. Briefly, the *Escherichia coli* strain BL21 (DE3) (Stratagene, La Jolla, CA, USA) was transformed with expression vector pET3a encoding WT α-syn and the bacteria were grown in LB medium to an optical density of 0.8. α-Syn expression was induced by 0.5 mM isopropyl β-d-1-thiogalactopyranoside for 3 h. The cells were lysed by sonication and the cell lysates were clarified by centrifugation at 14,000 × *g* for 30 min. α-Syn was precipitated by 50% ammonium sulfate at 4 °C. The solution was centrifuged at 4000 × *g* for 30 min at 4 °C and the resulting pellet was resuspended in 10 mM Tris pH 7.5. The solution was loaded onto a diethylaminoethyl cellulose column, which was eluted by a gradient of 0–500 mM NaCl, and the fractions containing α-syn (eluted at 200 mM NaCl) were heated to 75 °C for 20 min. Then, the solution was clarified by centrifugation at 14,000 × *g*, loaded onto a Superdex 75 HiLoad 26/60 column (GE Healthcare), equilibrated, and eluted in 50 mM Tris-HCl (pH 7.5) and 150 mM KCl. Pure α-syn (0.2–0.5 mM) in 50 mM Tris-HCl (pH 7.5) and 150 mM KCl was filtered through sterile 0.22 μm filters and stored at −80 °C. The α-syn concentration was determined spectrophotometrically using an extinction coefficient of 5960 M^−1^ cm^−1^ at 280 nm. For the fibril formation, α-syn was incubated at 37 °C under continuous shaking in an Eppendorf Thermomixer set at 600 r.p.m. The assembly was continuously monitored in a Cary Eclipse spectrofluorometer (Varian, Inc., Palo Alto, CA, USA) in the presence of Thioflavin T at an excitation wavelength of 440, an emission wavelength of 480, and an average time of 1 s.

### Immunohistochemistry

Brains post-fixed in 4% PFA were transferred to a 15% sucrose solution and then a 30% sucrose solution for gradient infiltration. The brains were cut into 40 μm-thick sections using a microtome (SM2010R, Leica, Germany). The slices were rinsed, immersed in a mixture of 3% H_2_O_2_ and methyl alcohol for 20 min to block endogenous peroxidase activity, and then blocked in 10% normal serum obtained from an animal of the same species that produced the secondary antibodies. The sections were incubated with rabbit anti-Iba1 (Wako, 1:800), mouse anti-tyrosine hydroxylase (TH, ImmunoStar, 1:2000), and Ser-129 phosphorylated α-syn antibody (Abcam, 1:2000) overnight at 4 °C, followed by rinsing three times with PBS. Then, the sections were incubated with a biotinylated secondary antibody against the primary antibodies for 2 h, followed by incubation with an ABC kit for 1 h. The sections were colorized with 3, 3′-diaminobenzidine (DAB) or alkaline phosphatase staining was performed using the Red Alkaline Phosphatase Substrate kit (SK-5100, Vector Labs, USA) according to the manufacturer’s instructions. Finally, the sections were mounted on glass slides. After dehydration and clearance with xylene, the sections were mounted with coverslips and observed under a microscope (DM4000B, Leica, Germany).

For paraffin-embedded tissues, paraffin sections (5 µm thick) were deparaffinized with xylene and then sequential decreasing concentrations of ethanol before going through the immunohistochemistry procedure described above.

### Perl’s-DAB ferric iron staining

The frozen sections were rinsed with distilled water for 10 min and a mixture of 2% K_4_[Fe(CN)_6_] (10016818, Sinopharm Chemical Reagent Co., Ltd, China) and 2% HCl was added dropwise to the sections. The sections were then incubated at room temperature for 30 min and washed three times with PBS. Then, eosin staining solution (C0109, Beyotime, China) was used to counterstain the cytoplasm, or the sections were counterstained with DAB for 3 min. After dehydration through ethanol and clearing in xylene, the sections were mounted on glass slides. The sections were examined under a Leica bright-field microscope (DM4000B, Leica, Germany). Blue or brown granules were regarded as positive staining of iron ions in the tissue section.

### Immunofluorescence

The brain sections were incubated with normal serum from the animal species that produced the secondary antibodies for 30 min and then incubated with mouse anti-transferrin (TF; Santa Cruz, 1:1000), mouse anti-TF receptor 1 (TFR1; Thermo, 1:500), rabbit anti-TF receptor 2 (TFR2; Abcam, 1:200), rabbit anti-ferroportin (FPn; Thermo, 1:1000), rabbit anti-ferritin (Abcam, 1:500), goat anti-Iba1 (Abcam, 1:200), and mouse anti-TH (ImmunoStar, 1:2000) overnight at 4 °C. Subsequently, the sections were incubated with DyLight 594-labeled goat anti-mouse IgG or goat anti-rabbit IgG. Finally, the sections were labeled with 4′ 6-diamidino-2-phenylindole (DAPI) (H-1500, Vector Labs, USA), mounted with anti-fluorescence quenching mounting medium, and observed under a confocal laser scanning microscope (SP8, Leica, Germany).

### Atomic absorption spectrometry

The brain tissue samples were accurately weighed with an analytical balance. The samples were treated with 200 μl of nitric acid (Sigma, purity ≥ 90%) at 100 °C for 30 min. Once the samples cooled to room temperature, they were diluted to 5 ml with 1% nitric acid. A flame atomic absorption spectrometer (ZEEnit700P, Analytik Jena, Germany) was used to detect the iron level.

### Stereological analyses of dopaminergic neurons in the substantia nigra

The stereology equipment consisted of a microscope (Olympus) with a controller that controls the field of view of the mirror to move precisely on the *X* and *Y* axes, a camera connected to a computer monitor, a pointer that accurately measures the depth of focus, a microcomputer (ProScan, UK), and a ×100 objective lens capable of performing accurate counting. Under the ×100 oil mirror, the stereological unbiased counting frame, the test frame, is superimposed on the tissue image. Stereological methods were used to investigate the number of neurons in the substantia nigra and, ventral tegmental area (VTA).

The total number of neurons in the substantia nigra region was calculated according to the following formula:

$${{N}}\left( {{\mathrm{Total}}} \right) = {\sum} {\mathrm{Q}} - \times 1/{\mathrm{ssf}} \times 1/{\mathrm{asf}} \times 1/{\mathrm{tsf}}$$

“ΣQ^−^” represents the total number of neurons in the substantia nigra; “ssf” represents the sampling score of the tissue section, i.e., 1/19; “asf” represents the area sampling fraction of the substantia nigra region within the tissue section, i.e., 2%; and “tsf” represents the thickness sampling fraction (H/T) of the substantia nigra region within the tissue section, where “T” is the average thickness of the tissue section (μm) and “H” is the test height of the optical stereo frame.

### Quantification and statistical analyses

To quantify the iron deposition in different brain regions and compare among different groups, we measured the optical density of the iron deposition in images taken under ×40 objective with Image-Pro Plus software. To quantify the appearance of iron-containing microglia in the double-labeled brain sections (after staining with iron dye K_4_[Fe(CN)_6_] and Iba1-immunohistochemistry), we randomly selected ten regions per section of the respective brain regions studied and counted the iron-staining Iba1-positive microglia against the total Iba1-positive microglia in the same area, and the proportion of the level of iron deposited in microglia was calculated. To quantify the change in the number of dopaminergic neurons containing iron-related proteins, we counted the number of dopaminergic neurons in the images taken under a ×20 objective where TH co-located with TF, TFR1, TFR2, or FPn, we randomly selected ten regions per section in the substantia nigra. For the quantification of TH-positive nerve terminals in the dorsolateral part of the putamen, we measured the intensities of the TH immunoreactivity in the images taken under ×40 objective and randomly selected ten regions per section. The results were presented as the mean values ± SEM. One-way analysis of variance (ANOVA) was used to evaluate the treatment groups compared with the control group. Tukey’s multiple comparisons test after two-way ANOVA was used to evaluate the difference between the left and right hemispheres using GraphPad Prism 8.0 software. A *p*-value < 0.05 was used throughout this study to indicate the significance.

## Results

### Iron deposition was increased in the nigra-striatal system after the α-syn PFFs treatment

**Fig. 1:**
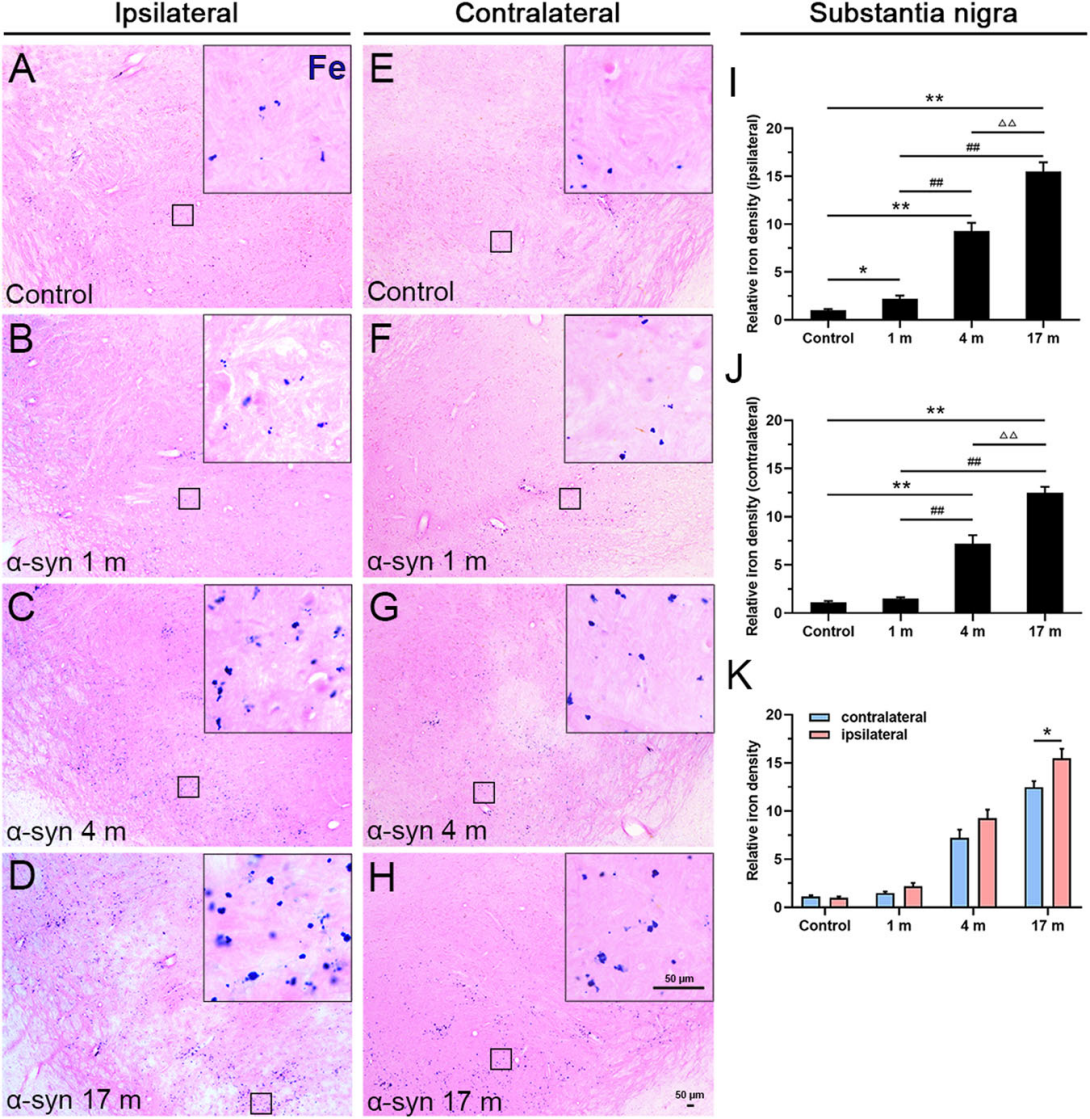
Ferric iron deposition in the substantia nigra. Chemical staining of iron ions showed a gradual increase in iron deposition, 1 (**B**), 4 (**C**), and 17 (**D**) months after the treatment with α-syn PFFs, compared to the control (**A**) in the ipsilateral side. **E**–**H** Chemical staining of iron ions in the control (**E**) and 1 (**F**), 4 (**G**), and 17 months (**H**) after treatment with α-syn PFFs in the contralateral side. The inset images depict the enlarged small boxes in **A**–**H**. **I**–**K** Quantitative analyses of the iron deposition density in the substantia nigra (ipsilateral side: **I**; contralateral side: **J**; both sides: **K**). Iron deposition staining levels were quantified with Image-Pro Plus software as the relative optical density above the background. α-Syn, α-synuclein. The data are presented as the mean ± SEM of three independent experiments. *n* = 3 in each group and time points. *p < 0.05 and **p < 0.01 vs. control group, ^##^*p* < 0.01 vs. 1-month group, ^ΔΔ^*p* < 0.01 vs. 4-month group, one-way ANOVA (**I**, **J**). *p < 0.05 vs. contralateral group, Tukey’s multiple comparisons test after two-way ANOVA (**K**). Scale bars: 50 μm in **A**–**H** and 50 µm in the insets.

Previous studies have shown that iron deposition is highly associated with dopaminergic neuron cytotoxicity and degeneration in PD patients and animal models^28–30^. To explore whether exogenously delivered α-syn PFFs treatment (via the olfactory epithelium) can alter iron deposition in the dopaminergic system, we first performed Perl’s chemical staining for ferric iron ion localization in monkey brain sections. As shown in Fig. 1A–H, we observed a robust increase in iron deposition in the substantia nigra in response to the α-syn PFFs treatment. Compared to the control (PBS treated) monkeys, the monkeys at 1 month after the treatment with α-syn PFFs exhibited significant increase of iron deposition in the ipsilateral (right) side of the substantia nigra, and the iron deposition severity increased significantly in a time-dependent manner from 1 to 17 months (Fig. 1I). Notably, we also detected the increased iron deposition in the contralateral side of the substantia nigra to α-syn PFFs delivery (Fig. 1J), whereas there were statistically significant differences between the two sides of the brain at 17 months after α-syn PFFs treatment (Fig. 1K).

**Fig. 2:**
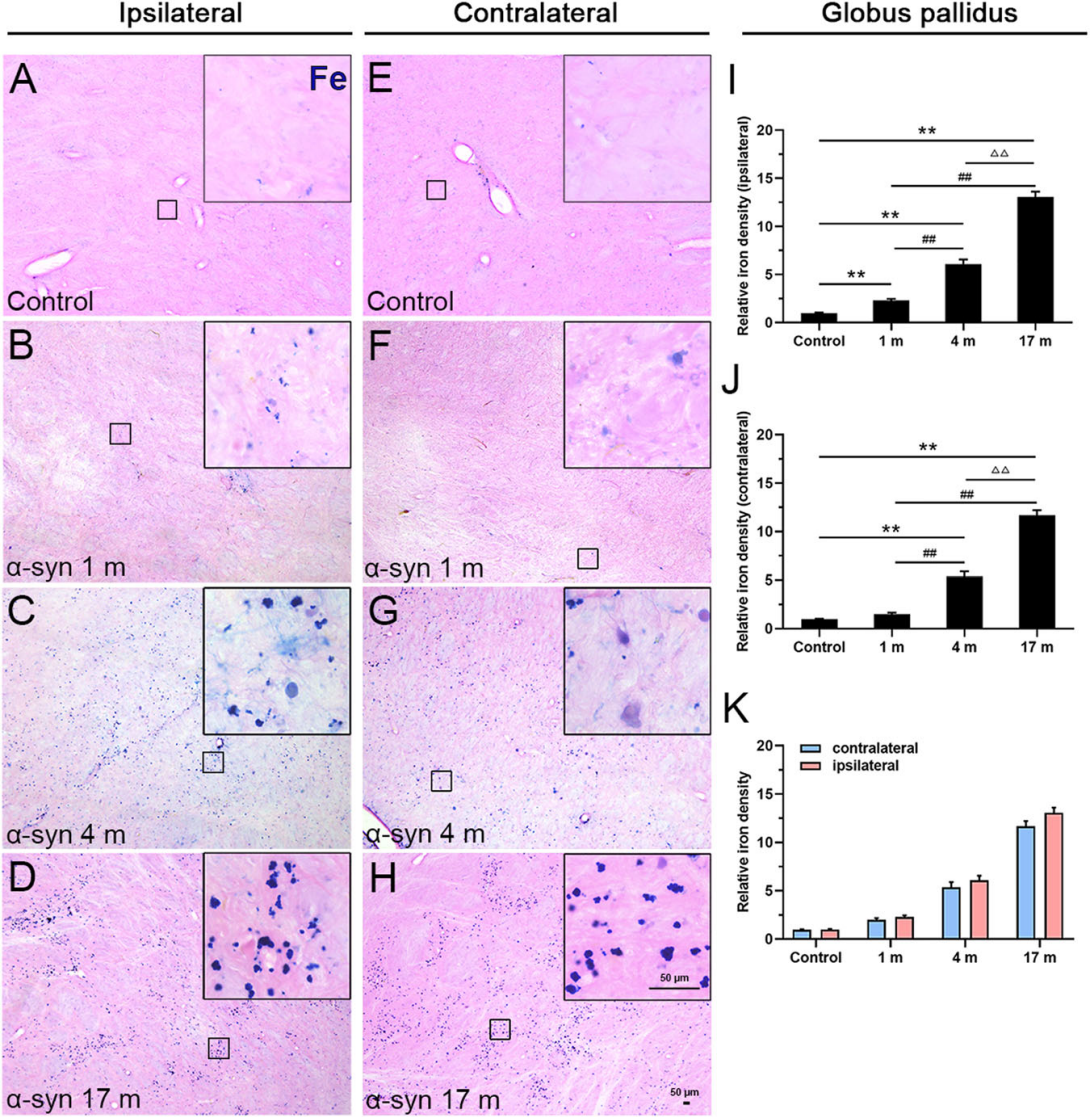
Ferric iron deposition in the globus pallidus. **A**–**D** Chemical staining of iron ions showed a gradual increase in iron deposition, 1 (**B**), 4 (**C**), and 17 months (**D**) after the α-syn PFFs treatment, compared to the control (**A**) in the ipsilateral side. **E**–**H** Chemical staining of iron ions in the control (**E**) and 1 (**F**), 4 (**G**), and 17 months (**H**) after the treatment with α-syn PFFs in the contralateral side. The inset images depict the enlarged small boxes in **A**–**H**. **I**–**K** Quantitative analyses of the iron deposition density in the globus pallidus (ipsilateral side: **I**; contralateral side: **J**; both sides: **K**). α-Syn, α-synuclein. The data are presented as the mean ± SEM of three independent experiments. **p < 0.01 vs. control group, ^##^*p* < 0.01 vs. 1-month group, ^ΔΔ^*p* < 0.01 vs. 4-month group, one-way ANOVA (**I**, **J**). Scale bars: 50 μm in **A**–**H** and 50 µm in the insets.

As shown in Fig. 2, consistent changes similar to those in the substantia nigra were observed in the globus pallidus (Fig. 2A–J), but there was no statistically significant difference between the ipsilateral and contralateral sides of the brain (Fig. 2K). Moreover, we detected that the increasing tendency of iron deposition in the caudate nucleus (Supplementary Fig. 2a) was similar to that in the globus pallidus, but the degree was very mild. Interestingly, there was no obvious iron deposition in the hippocampus (Supplementary Fig. 2b), entorhinal cortex (Supplementary Fig. 2c), and olfactory bulb (Supplementary Fig. 2d), compared with the control monkeys after α-syn PFFs treatment.

It was well-known that Perl’s chemical staining visualized the ferric iron, as shown in Figs. 1 and 2, to examine the presence and potential changes of ferrous iron in response to α-syn PFFs treatment, we performed Turnbull staining. It appeared that the staining intensity of ferrous iron in the substantia nigra and globus pallidus was very weak, and mainly in the tissues around blood vessels, and there was no clear increase in response to α-syn PFF treatment (Supplementary Fig. 3a, b).

### Iron contents in different brain regions after the α-syn PFFs treatment

**Fig. 3:**
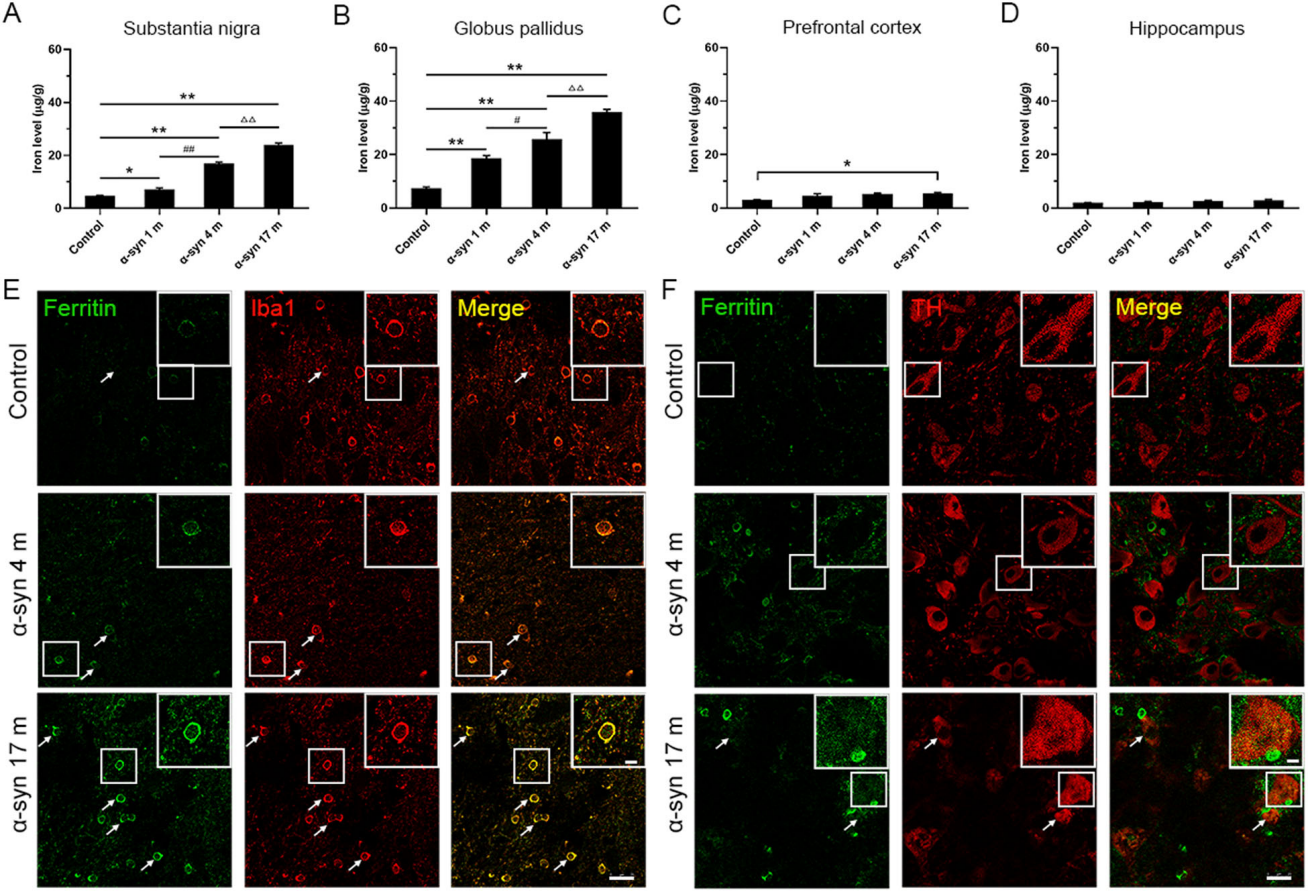
Iron contents in different brain regions and ferritin in microglia and dopaminergic neurons. The iron content in the substantia nigra (**A**), globus pallidus (**B**), prefrontal cortex (**C**), and hippocampus (**D**) was determined by AAS. **E** Colocalization of ferritin and Iba1 in the substantia nigra. **F** Colocalization of ferritin and TH in the substantia nigra. The data are presented as the mean ± SEM of three independent experiments. α-Syn, α-synuclein. *p < 0.05 and **p < 0.01 vs. control group, ^#^*p* < 0.05 and ^##^*p* < 0.01 vs. 1-month group, ^ΔΔ^*p* < 0.01 vs. 4-month group, one-way ANOVA. Scale bars: 25 μm in **E** and **F**, and 25 µm in the insets.

To further validate the changes of iron contents in different brain regions following different treatments, we selected the substantia nigra, globus pallidus, prefrontal cortex, and hippocampus, and used atomic absorption spectrometry (AAS) to measure the iron content. Compared to the control monkeys, it was evident that the iron content increased by one- to twofolds in the substantia nigra and globus pallidus in the monkeys treated with the α-syn PFFs, and the iron content increased in a time-dependent manner from 1 to 17 months (Fig. 3A, B). There were no statistical differences in the iron content of hippocampus between α-syn PFFs treatment and control (Fig. 3D), whereas the iron content in the prefrontal cortex of monkeys treated with α-syn PFFs for 17 months was significantly increased compared with the control (Fig. 3C). The AAS data are consistent with the observations of the chemical iron staining. In addition, we observed that the expression of ferritin in the substantia nigra of α-syn PFF-treated monkeys was more than that of the control monkey in a time-dependent manner. Interestingly, ferritin was mainly localized in microglia (Fig. 3E), the expression of ferritin in the dopaminergic neurons was low, even though it was colocalized with TH in the 17-month monkeys after α-syn treatment (Fig. 3F).

### Selectively increased iron deposition in microglia

**Fig. 4:**
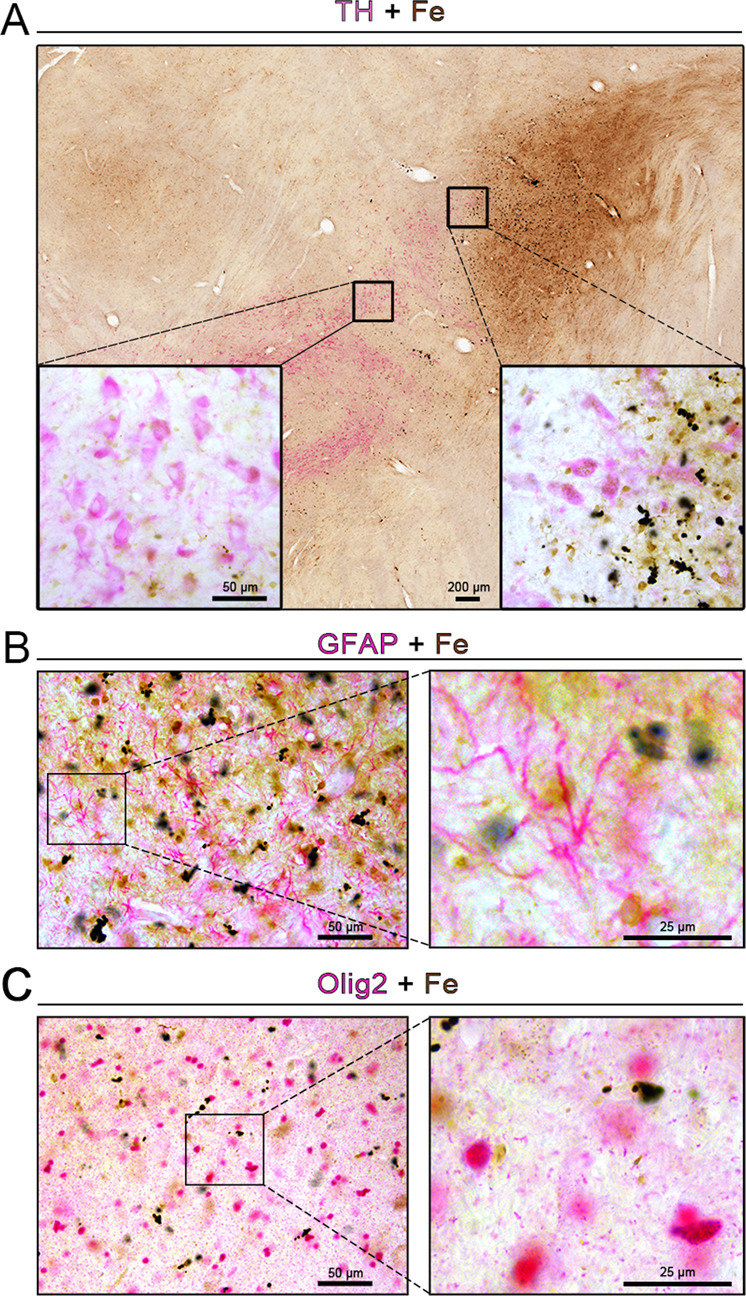
Cellular localization of iron deposits in the substantia nigra. **A**–**C** Double-labeling of chemically stained iron ions (dark brown) along with immunohistochemically stained with TH (**A**), GFAP (**B**), or Olig2 (**C**) antibody (magenta) showed heavy iron deposition 17 months after treatment with α-syn PFFs. The inset images depict the enlarged small boxes in **A**–**C**. Scale bars: 200 μm in **A** and 50 µm in the inset; 50 µm in **B**, **C**, and 25 µm in the enlarged images.

**Fig. 5:**
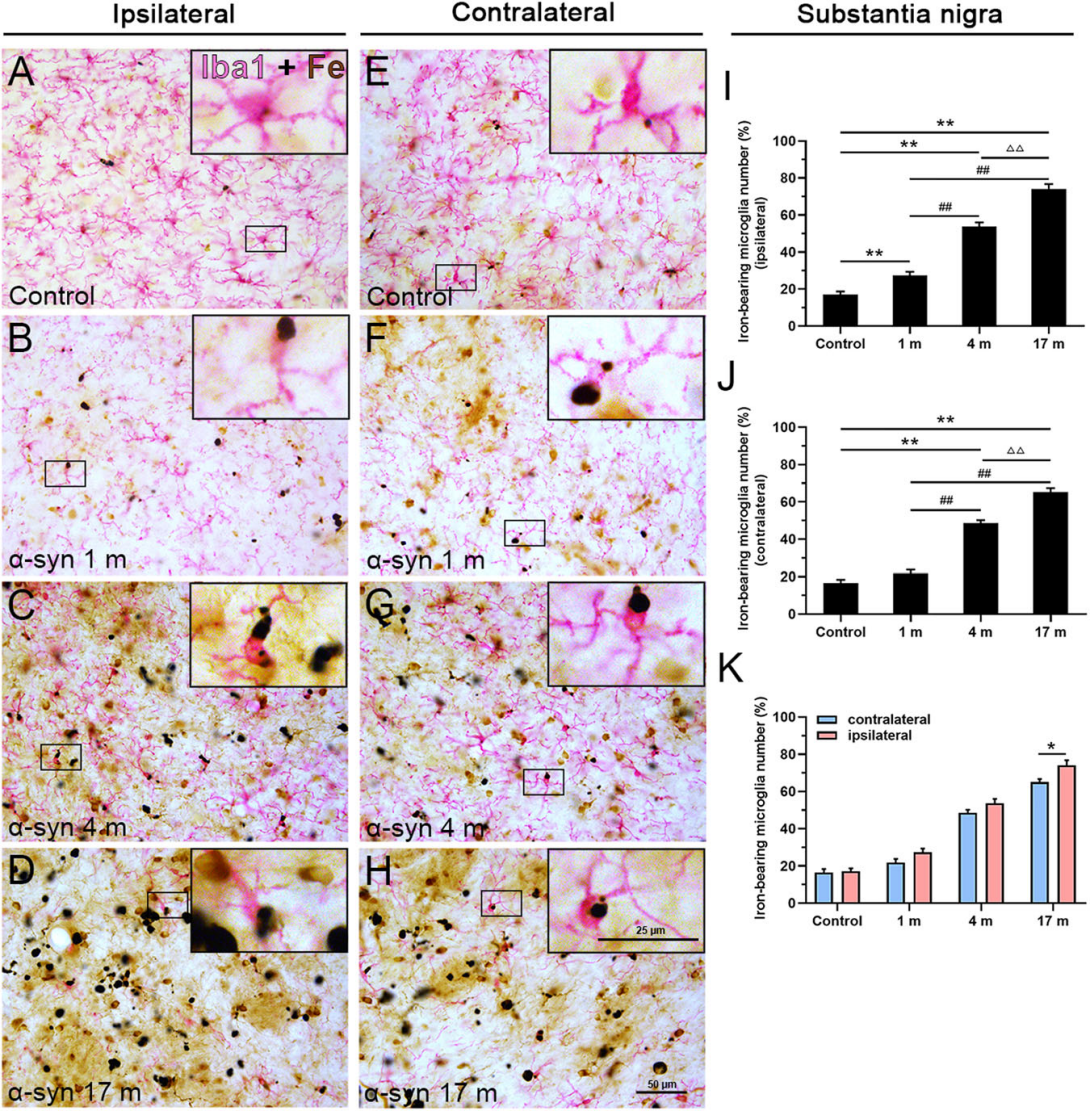
Cellular localization of iron deposits in microglia of the substantia nigra. Double-labeling of chemically stained iron ions (dark brown) along with immunohistochemical staining with an Iba1 antibody (magenta) showed overlapping of iron deposition on Iba1-positive microglia in the control group (**A**) and 1, 4, and 17 months after the treatment with α-syn PFFs in the ipsilateral side (**B**–**D**), control group (**E**), and 1, 4, and 17 months after treatment with α-syn PFFs in the contralateral side (**F**–**H**). **I**–**K** Quantitative analyses of iron deposition in microglia in the substantia nigra (ipsilateral side: **I**; contralateral side: **J**; both sides: **K**). α-Syn, α-synuclein. The data are presented as the mean ± SEM of three independent experiments. **p < 0.01 vs. control group, ^##^*p* < 0.01 vs. 1-month group, ^ΔΔ^*p* < 0.01 vs. 4-month group, one-way ANOVA (**I**, **J**). *p < 0.05 vs. contralateral group, Tukey’s multiple comparisons test after two-way ANOVA (**K**). Scale bars: 50 μm in **A**–**H** and 25 µm in the insets.

**Fig. 6:**
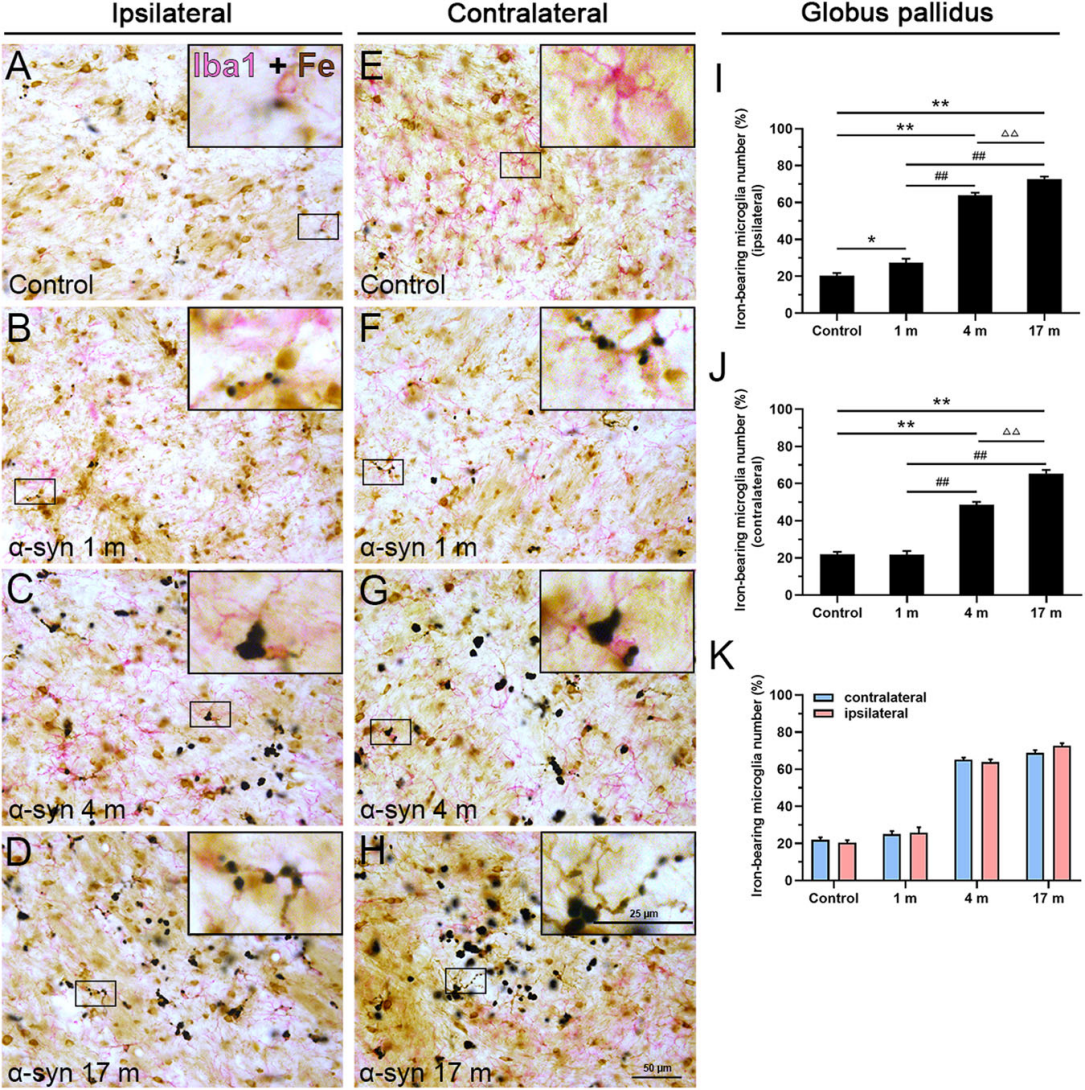
Cellular localization of iron deposits in microglia in the globus pallidus. Double-labeling of chemically stained iron ions (dark brown) along with immunohistochemical staining using an Iba1 antibody (magenta) shows overlapping of iron deposition in Iba1-positive microglia in the control (**A**) and 1, 4, and 17 months after the treatments with α-syn PFFs in the ipsilateral side (**B**–**D**), control group (**E**) and 1, 4, and 17 months after treatment with α-syn PFFs in the contralateral side (**F**–**H**). **I**–**K** Quantitative analyses of iron deposition in microglia in the substantia nigra (ipsilateral side: **I**; contralateral side: **J**; both sides: **K**). α-Syn, α-synuclein. The data are presented as the mean ± SEM of three independent experiments. *p < 0.05 and **p < 0.01 vs. control group, ^##^*p* < 0.01 vs. 1-month group, ^ΔΔ^*p* < 0.01 vs. 4-month group, one-way ANOVA (**I**, **J**). Scale bars: 50 μm in **A**–**H** and 25 µm in the insets.

It has been shown that excess iron is toxic to dopaminergic neurons^31,32^. Next, we examined the cell-type-specific localization of iron deposition, e.g., where the iron deposition was the most enriched. As shown by the chemical staining of iron ions in the substantia nigra (Fig. 1A–H), the level of iron was increased in response to α-syn PFFs treatment. Surprisingly, the double-labeling by immunostaining for TH, which is the key enzyme involved in dopamine synthesis, and the chemical staining of iron ions showed limited or no overlap (colocalization) between TH and iron deposits in dopaminergic neurons, iron deposition mainly exists in the dorsolateral area of the substantia nigra (Fig. 4A). Iron deposits were not localized in astrocytes (GFAP-positive) (Fig. 4B), nor in oligodendrocytes (Olig2-positive) (Fig. 4C). In contrast, we observed that many microglia in the substantia nigra (Fig. 5A–H) and globus pallidus (Fig. 6A–H) exhibited robust iron deposition. On the ipsilateral side, iron deposition significantly increased 1 month after α-syn PFFs treatment compared to the control group, and the iron deposition in microglia severity increased further in a time-dependent manner from 1 to 17 months (Figs. 5I and 6I). Moreover, we detected that the extent of the increase in iron deposition in microglia in the substantia nigra and globus pallidus in the contralateral side to the α-syn PFFs delivery (Figs. 5J and 6J) was similar to that in the ipsilateral side (Figs. 5I and 6I). In the 1-month treatment group, the increase of iron deposition in the contralateral side was not statistically significant in microglia in the substantia nigra and globus pallidus. Moreover, the iron deposition increased to a large extent in the ipsilateral side than that in the contralateral side of the substantia nigra at 17 months after α-syn PFFs treatment (Fig. 5K), but there was no statistical difference between the ipsilateral and contralateral sides of the globus pallidus (Fig. 6K). In comparison, microglia in other brain regions, such as the caudate nucleus (Supplementary Fig. 4a), putamen (Supplementary Fig. 4b), prefrontal cortex (Supplementary Fig. 4c), and hippocampus (Supplementary Fig. 4d), exhibited much less iron deposition. These data indicate that iron deposition is enriched specifically in the substantia nigra and globus pallidus and at the cellular level, specifically in the microglia.

### Expression of iron-related proteins in dopaminergic neurons

**Fig. 7:**
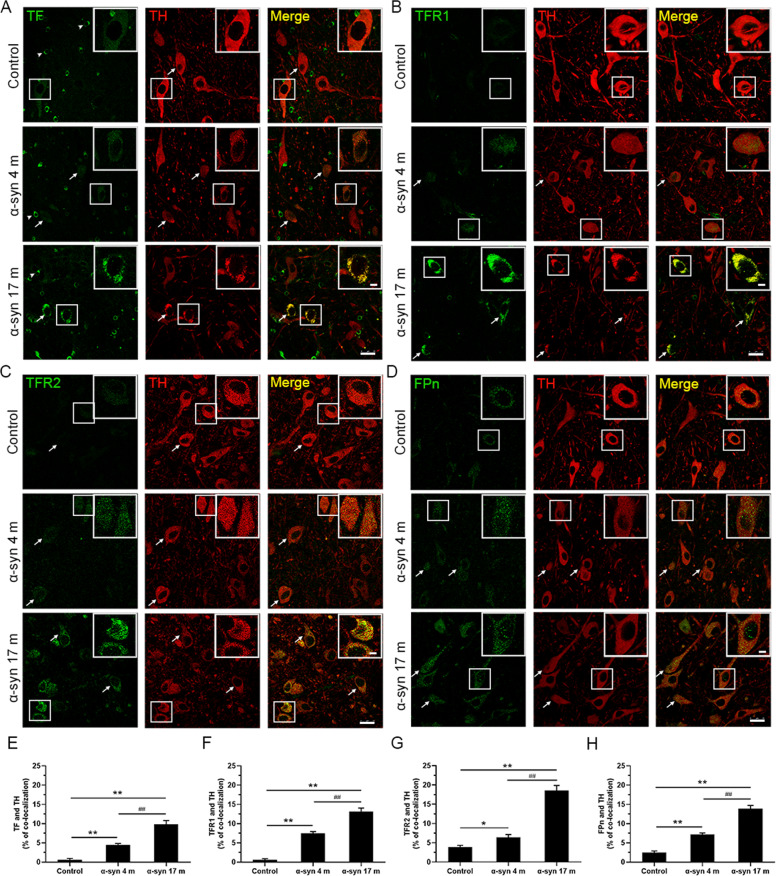
Double fluorescence images showing the colocalization of TF, TFR1, TFR2, FPn, and TH in dopaminergic neurons in the substantia nigra. **A**–**D** Immunofluorescence intensity of the TF-positive (**A**), TFR1-positive (**B**), TFR2-positive (**C**), and FPn-positive (**D**) signal increased, while the TH-positive signal decreased 4 and 17 months after α-syn PFFs treatments compared to the control. Arrows show colocalization of TF, TFR1, TFR2, FPn with TH in dopaminergic neurons. Arrowheads (**A**) show the presence of TF in oligodendrocytes. **E**–**H** Colocalizations of TF, TFR1, TFR2, FPn, and TH increased in the substantia nigra after α-syn PFFs treatment. α-Syn, α-synuclein; TF, transferrin; TFR1, transferrin receptor 1; TFR2, transferrin receptor 2; FPn, ferroportin. The data are presented as the mean ± SEM of three independent experiments. *p < 0.05 and **p < 0.01 vs. control group, ^##^*p* < 0.01 vs. 4-month group, one-way ANOVA. Scale bars: 25 μm in **A**–**D**.

As iron deposition increased in the substantia nigra but the deposits were mostly localized in microglia rather than dopaminergic neurons, we examined how microglia with a high iron content might affect dopaminergic neurons. Using double immunofluorescence staining with antibodies specifically against iron-related proteins and TH, we observed altered expression of iron-related proteins in dopaminergic neurons. Moreover, TF, TFR1, TFR2, and FPn all partially colocalized with TH (Fig. 7A–D). Compared with the control group, iron-related proteins in dopaminergic neurons in the substantia nigra appeared increased at the 4- and 17-month time points after α-syn PFFs treatments and many TH-positive cells were also positive for TF (Fig. 7E), TFR1 (Fig. 7F), TFR2 (Fig. 7G), and FPn (Fig. 7H).

### No dopaminergic neuronal loss in the substantia nigra, but neurodegenerative terminals in the putamen

**Fig. 8:**
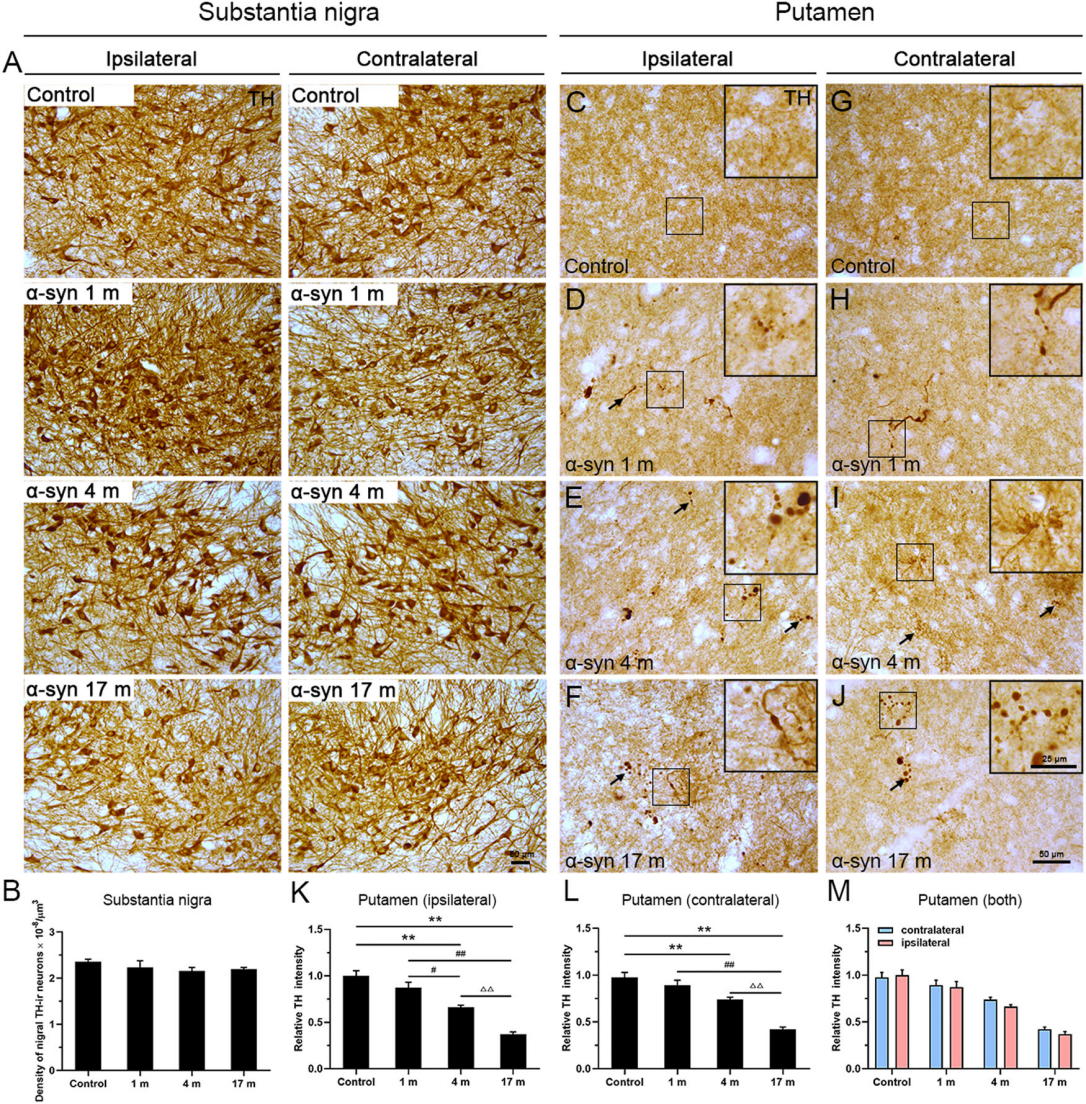
Immunohistochemical images showing TH-positive dopaminergic neurons and terminals in the substantia nigra and putamen. **A** Dopaminergic neurons appeared to have a normal morphology without clear degenerative alterations in different groups. **B** Stereological analyses of dopaminergic neurons in the substantia nigra. **C**–**J** Scattered dystrophic axonal swellings were observed in the putamen on both sides, at different time points in response to α-syn PFFs treatment. **C**, **G** In comparison, the control displays homogenous distribution with no pathological terminals. **K**–**M** Quantitative analyses of the intensity of TH immunoreactivity in the putamen (ipsilateral side: **K**; contralateral side: **L**; both sides: **M**). α-Syn, α-synuclein; TH, tyrosine hydroxylase. The data are presented as the mean ± SEM of three independent experiments. **p < 0.01 vs. control group, ^#^*p* < 0.05, ^##^*p* < 0.01 vs. 1-month group, ^ΔΔ^*p* < 0.01 vs. 4-month group, one-way ANOVA (**K**, **L**). Scale bars: 50 μm in **A**, **C**–**J** and 25 µm in the insets.

Dopaminergic neuronal cell loss and α-syn aggregation in the substantia nigra are characteristic features of PD. To examine effects of dopaminergic neurons in response to α-syn PFFs administrations via the olfactory epithelium, we quantified dopaminergic neurons in the substantia nigra (Fig. 8A, B) and nerve terminals in the putamen (Fig. 8C–M). Stereological quantitative analyses showed no difference in number of dopaminergic neurons among the different groups (Fig. 8B). Very interestingly, a progressive loss of dopaminergic (TH-positive) terminals was detected in the dorsolateral part of the putamen (Fig. 8K, L). The overall density of TH-positive terminals was progressively reduced, and many of the terminals showed signs of damage, expressed as TH-containing axonal swellings (arrows in Fig. 8D–J). Likewise, there was no statistical difference between the ipsilateral and contralateral sides (Fig. 8M).

We, at last, examined whether treatment with α-syn had any effects on α-syn aggregation and phosphorylation. We barely observed any phosphorylated (pS129) α-syn-positive neurons in the substantia nigra of the control (PBS) group (Supplementary Fig. 5a), but we detected sparsely distributed pS129-α-syn-positive neurons in the substantia nigra in response to α-syn PFFs treatment (Supplementary Fig. 5b–d). α-Syn immunoreactivity appeared in diffuse pattern, no typical LB nor Lewy neurites were detected (Supplementary Fig. 5b–d). No distinct phosphorylated α-syn-positive neurons nor terminals were observed in the putamen in the 1-, 4-, and 17-month groups after the administration of α-syn PFFs (Supplementary Fig. 5f–h), which was similar to the control group (Supplementary Fig. 5e). Moreover, we also performed immunohistochemical staining with α-syn or phosphorylated α-syn antibodies and also co-stained with iron chemical (Perl’s) staining to examine whether α-syn colocalize with iron deposition in the substantia nigra. It was evident that iron depositions were largely present in the microglia-like cells (as shown Figs. 5 and 6), whereas α-syn appeared as fine granules reflecting a localization in nerve terminal structures. We only observed little overlapping between α-syn and iron deposition (Supplementary Fig. 6a). Interestingly, we observed to a certain extent overlapping between phosphorylated α-syn and iron deposition in the 4- and 17-month group after α-syn PFF treatments (Supplementary Fig. 6b).

### No behavioral alterations after the α-syn PFFs treatment

To examine whether the α-syn PFFs treatment may induce behavioral changes, we performed two types of behavioral tests. One was the delay match-to-sample (DMTS), which evaluated the short term memory of cognitive behavior. The other was the pick-up test (PUT), which quantitatively measured the fine hand motor performance skills. Due to the long-term (for 6 months) training process, we only performed the two tests in the monkeys 17 months after α-syn PFFs delivery. Interestingly, we did not observe significant alterations on either DMTS (Supplementary Fig. 7a, b) or PUT tests (Supplementary Fig. 7c, d).

## Discussion

Iron accumulates to a much higher extent in specific brain regions in various neurodegenerative diseases^33–36^. The functional impairment of dopaminergic neurons in the nigra-striatal system caused by high iron in the brain has drawn considerable attention in the field of PD research^37,38^. Dopaminergic neuron degeneration is evident in the brains of PD patients and iron accumulation is usually associated with this cytopathic effect^39,40^. Here we present data confirming the presence of excessive iron deposition in specific brain regions in monkeys, mimicking the human situation.

During the course of PD, dopaminergic neurons gradually lose their function, eventually leading to cell death. Iron plays a key role in this process; thus, the excessive deposition of iron may be involved in the degenerative death process of dopaminergic neurons in PD^32,41,42^. In the present study, up to the 1, 4, and 17 months after the treatment with the α-syn PFFs, we did not observe dopaminergic neuron loss in the substantia nigra, nor motor and cognitive dysfunction (Supplementary Fig. 7) in consistent with the previous report^43^, but rather, only detected signs of dopaminergic neurodegeneration, i.e., reduced TH intensity and axonal swelling in the putamen^44–46^. Interestingly, an increase in the iron content was detected in the brains of monkeys in the PD model groups and further studies revealed that the increased iron was mainly deposited in the substantia nigra and globus pallidus, which are also the brain regions that exhibit lesions in PD patients^47–51^. These results provide evidence supporting the tropism of α-syn PFFs in the substantia nigra^52–54^, which leads us to speculate that iron deposition precedes dopaminergic neuron loss. However, further investigation on the underlying mechanisms on how α-syn aggregates causes PD and the relationship between α-syn aggregation and iron metabolism is warranted. In fact, some human PD brain regions, such as the substantia nigra and striatum, exhibit excessive iron accumulation^55,56^. Similarly, iron deposits are present in the substantia nigra in 6-OHDA-induced PD rat models^57,58^. In addition, methyl-4-phenyl-1, 2, 3, 6-tetrahydropyridine, a neurotoxin, can induce PD-like symptoms and iron is involved in its conversion to the toxic metabolite 1-methyl-4-phenylpyridinium ion^59^; during conversion, the induced cellular damage and neurotoxicity are considered to be associated with changes in iron and iron metabolism^60^. Importantly, α-syn contains divalent metal ions, including iron-binding sites^61^. Thus, it is conceivable that exposure to α-syn PFFs may cause iron deposition, at least partially, by altering iron metabolism in the brain areas affected in PD, eventually resulting in dopaminergic neuron damage.

The reasoning proposed here is supported by Oakley et al.^16^, who examined fresh specimens of the substantia nigra from PD patients using sensitive and specific wavelength dispersive electron probe X-ray microanalysis coupled with cathodoluminescence spectroscopy, and found that the iron content in the neurons in the substantia nigra was increased, suggesting that increased neuronal iron in PD may be a primary event leading to α-syn aggregation. In the present study, we observed that iron mostly accumulates in microglia rather than in dopaminergic neurons. Furthermore, dopaminergic neuron death was not detected in the monkeys treated with α-syn PFFs. Although impaired microglia have been reported to be closely associated with iron deposition^62,63^, in the present study, most microglia in this region were activated. The excessive activation of microglia could cause neurotoxicity and produce numerous neuroimmune proinflammatory factors that lead to oxidative stress, further promoting dopaminergic neuron damage^41,64^.

The increased iron concentration in PD brains may be due to the abnormal regulation of iron metabolism in neurons and glia^65^. TF is an important iron-binding, transport and storage protein and an important regulatory protein of iron metabolism in the body. Both TF and TFR are involved in maintaining the balance of iron metabolism, and iron transport by TF/TFR is the main source of iron in tissues. An increase in TF/TFR leads to an increase in the intracellular iron content^13^. It has been demonstrated by experimental approaches that the TF/TFR2 complex may have an important role in the etiology of PD^66,67^. Currently, FPn is the only known transmembrane iron transporter and FPn dysfunction causes disturbances in iron metabolism^68^. In the present study, an increase in the TF/TFR1/TFR2/FPn proteins was detected in the substantia nigra, leading to an increase in the intracellular iron ion content and iron accumulation^13,68^. Moreover, we observed cell-type-dependent iron enrichment in microglia and low iron abundance in dopaminergic neurons in the substantia nigra. Microglia maintain iron homeostasis in the brain mainly by sequestering and storing iron within ferritin^69^, and microglia activation is an important cause of high iron concentrations in the brain^70^. As expected, we also detected an increase in the immunoreactivity of ferritin in α-syn PFFs-treated microglia, which is related to the pathogenesis of degeneration of nigrostriatal dopamine neurons in PD^18^. Therefore, it is conceivable that the iron metabolism-related proteins TFR/TF and FPn modulate iron transport between different types of cells in PD-affected brain regions in response to α-syn PFFs treatment, which could impair the normal transport of iron by iron-related proteins in the nigra-striatal system, and the iron deposition in microglia may indirectly cause dysfunction in dopaminergic neurons in PD^71,72^.

What is the consequence of increased iron deposition in microglia rather than dopaminergic neurons in the monkeys treated with α-syn PFFs treatment? Although we did not observe clear dopaminergic neuronal loss, we detected an increased cellular level of iron-related proteins, such as TFR1/TFR2/TF and FPn, in dopaminergic neurons. Therefore, we propose two possible underlying mechanisms. First, exposure to α-syn PFFs treatment may initiate the process of neuroinflammation by triggering iron accumulation in microglia and then inducing cascade responses between iron deposition and microglial activation, generating hydrogen peroxide and hydroxyl radicals, releasing proinflammatory factors and leading to α-syn aggregation, dopaminergic neuron toxicity, and degeneration^22,73^. Second, under both physiological and pathophysiological conditions, iron continually moves across neurons, microglia, and astrocytes^74^. Although it has been shown that the level of iron is elevated in dopaminergic neurons in PD^16^, we did not observe a similar phenomenon in dopaminergic neurons, but we detected robust iron deposits in microglia. Two reasons may explain this inconsistency. Most, if not all, studies using PD patient materials were obtained from patients with an advanced stage of PD, while the current monkey model treated with α-syn PFFs treatment only mimicked the early stage and even the prodromal stage of PD. It is conceivable that iron deposition first appears in microglia in the substantia nigra, and then, with the progression of PD pathology, the iron in microglia may translocate from nearby microglia to dopaminergic neurons and be regulated by iron-related proteins present in dopaminergic neurons. Moreover, no dopaminergic neuronal loss in the substantia nigra, decreased TH immunoreactivity in the putamen, together with mildly and sparsely distributed phosphorylated α-syn in dopaminergic neurons in the substantia nigra, but absence in the putamen reinforced the claim that iron deposition in microglia precedes dopaminergic neuronal degeneration. In summary, our findings indicated that iron deposition in the nigra-striatal system of monkeys precedes dopaminergic neuronal degeneration after nasal administration of exogenous α-syn PFFs. Based on the present study, the iron deposition in microglia and abnormal iron transport-related proteins appear to involve in the pathogenesis of PD and may become a tool for the early diagnosis of PD, such as magnetic resonance imaging of iron contents. The modulation of abnormal iron metabolism may be a potential therapeutic strategy for PD.

## Supplementary information

supplementary information

Supplementary Fig. 1

Supplementary Fig. 2

Supplementary Fig. 3

Supplementary Fig. 4

Supplementary Fig. 5

Supplementary Fig. 6

Supplementary Fig. 7

Supplementary Fig. 8
