# Supplementary material for: Intranasal administration of α-synuclein preformed fibrils triggers microglial iron deposition in the substantia nigra of Macaca fascicularis
Source: Cell Death Dis. 2021 Jan 13;12(1):81. doi: 10.1038/s41419-020-03369-x (PMC7807015; doi:10.1038/s41419-020-03369-x)
Supplement: Supplementary file 1 — supplementary information [file 41419_2020_3369_MOESM1_ESM.docx]

**Supplementary information:**

Guo JJ et al.

**Materials and methods**

**Turnbull ferrous iron staining**

The frozen sections were rinsed with distilled water for 5 min, and the Turnbull staining kit was used to detect ferrous iron in the tissue according to the instructions in the kit (GMS80047.5, GENMED, USA). The sections were incubated at room temperature for 30 min and washed three times with distilled water. Then, eosin staining solution (C0109, Beyotime, China) was used to counterstain the cytoplasm. The sections were dehydrated with ethanol and cleared in xylene and they were then mounted on glass slides. The sections were examined under a Leica brightfield microscope (DM4000B, Leica, Germany). Blue granules were regarded as positive staining of ferrous iron ions in the tissue section.

**Behavioral tests**

***Delayed match-to-sample (DMTS)***

Each (control or treated) monkey was subjected to the delayed match-to-sample (DMTS) tasks to evaluate the short term memory of cognitive behavior, which was tested using a panel modified in terms of the Wisconsin General Testing Apparatus (WGTA) principle.

The panel for DMTS tasks has two arm portals, with three reward food wells in the main panel. The panel used for the DMTS task has three arm portals in the mainframe, and three reward food wells on a testing tray that are perpendicularly attached to the mainframe, each food well is covered with an identical opaque Lexan sliding access door stuck with one specific object. The animals were trained to operate the panel, which was attached to the doorway of the testing-cage. The training started with allowing the animal to push the middle sliding access door on which there was an object (cue) attached, allowing the animals to retrieve a reward food item in the food well. Once the animals were regularly skilled in pushing the middle sliding door away and taking treats from the middle food well, they were trained to operate the left or right sliding access doors and retrieve showed the food items from the food well under the access door. One door bore the cue, and one door showed a novel object. If the animal chose to push the access door with the cue objects and retrieved the food item, the trial was considered correctly completed; otherwise, it was considered incorrectly completed. When the animal could complete 30 trials with a success rate of 85% without any delay, the animal was considered ready for formal testing. For each test session, four variable time delays 5, 15, 30 and 60 s, it means the time between the presentation of the cue sample and the comparison stimuli. The four delay testing was randomly set to perform 30 trials each daily testing session, and the percentage of correct responses was measured in two groups (n = 3 in control (PBS) group; n = 3 in α-syn treatment group, 17 month after α-syn delivery). The data are presented as the mean ± SEM. Student's *t*-test and two-way ANOVA were performed with Prism 8.0 software.

***Motor behavioral assessments: the pick-up test (PUT)***

We used Pick-up Test (PUT) to quantitatively measure fine hand motor performance skills. The time for the monkeys to pick up all six pieces of dried sweet potato without interruption from the nine-well panel was measured as a major index to evaluate fine hand motor function. On the testing day, the monkeys were deprived of food, then transferred to the behavior cage equipped with a pick-up test panel. The panel was divided into left and right sides. Six pieces of dried sweet potato were placed freely in the nine holes. Monkeys were trained to pick up the dried sweet potato pieces on the platform one by one with their left hand through the right hole. We defined "success" in the task as occurring only when the monkey sequentially picked up all six dried sweet potato pieces one by one with its left hand. The time from the appearance of the monkey's hand at the hole to the disappearance after picking up all six dried sweet potato pieces was defined as the time required for completion of one session of PUTs. The measurement was performed twice a month. The monkeys performed seven tasks altogether, and each task was repeated six times for each hand. Thus, the total time was an average of seven testing days, which included daily data that were averaged from six trials on each side. These data are reported as the mean ± SEM. Student's *t*-test and two-way ANOVA were performed with Prism 8.0 software.

**Supplementary Figure Legends**

**Supplementary Fig. 1** **Schematic drawing illustrating the experimental design and treatments.**

**Supplementary Fig. 2 Iron deposition in different brain regions.** Iron deposition in the caudate nucleus (**a**), the hippocampus (**b**), the entorhinal cortex (**c**) and in the olfactory bulb (**d**). Chemical staining of iron ions showed very low levels of iron deposition in these brain regions regardless of the α-syn PFFs treatment, except for 4 and 17 months after the α-syn PFFs treatment in the caudate nucleus. Scale bars: 50 μm.

**Supplementary Fig. 3 Ferrous iron in the substantia nigra** (**a**) **and globus pallidus** (**b**). Ferrous iron (blue), eosin staining solution counterstain the cytoplasma (red). The inset images depict the enlarged small boxes in **a**-**b**. Scale bars: 50 μm in **a**-**b**; and 25 µm in the insets.

**Supplementary Fig. 4 Cellular localization of iron deposits in microglia.** Iron deposits in microglia in the caudate nucleus (**a**), the putamen (**b**), the prefrontal cortex (**c**) and the hippocampus (**d**). The inset images depict the enlarged small boxes in **a**-**d**. Scale bars: 50 μm in **a**-**d**; and 25 µm in the insets.

**Supplementary Fig. 5 Immunohistochemical images showing S129 phosphorylated α-syn in the substantia nigra and putamen of monkeys.** **a**-**d** In contrast to the control, weakly and diffusely stained phosphorylated α-syn profiles appeared in the 1-month, 4-month, and 17-month groups after treatment with α-syn PFFs. **e**-**h** In contrast to the control, no distinctly stained phosphorylated α-syn profiles were observed in the 1-month, 4-month and 17-month groups after the treatment with α-syn PFFs in the putamen. α-Syn, α-synuclein. Scale bars: 50 μm in **a**-**h**.

**Supplementary Fig. 6 Co-localization of iron deposition with α-syn or S129 phosphorylated α-syn in the substantia nigra. a**-**b** Double labeling of chemically stained iron ions (dark brown) along with immunohistochemical staining using α-syn (**a**) and S129 phosphorylated α-syn antibody (**b**) (Magenta). Little overlapping between α-syn/phosphorylated α-syn and iron deposition was observed in control and 1 month after α-syn PFF treatments, but more rubust in the 4 and 17-months groups. Scale bars: 50 μm in **a**-**b**; and 25 µm in the insets.

**Supplementary Fig. 7 Behavioral tests in response to α-syn PFF treatments**

**a**-**b** The short term memory test (DMTS): After 4 months of training, the monkeys 17 month after treatments with α-syn PFF or PBS did not any significant difference in average food pick-up time and different intervals. **c**-**d** The fine hand motor performance test (PUT): No difference was observed in time required to pick up food for both right and left hands, respectively (**c**) or pulled together (**d**).
