# Supplementary figures and images for: Intranasal administration of α-synuclein preformed fibrils triggers microglial iron deposition in the substantia nigra of Macaca fascicularis
Source: Cell Death Dis. 2021 Jan 13;12(1):81. doi: 10.1038/s41419-020-03369-x (PMC7807015; doi:10.1038/s41419-020-03369-x)

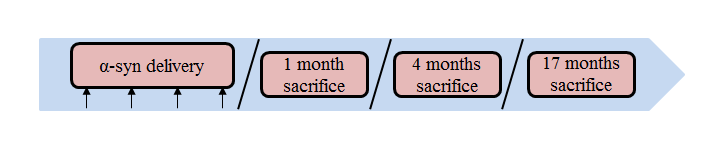

Supplement: Supplementary file 2 — Supplementary Fig. 1 [file 41419_2020_3369_MOESM2_ESM.tif]

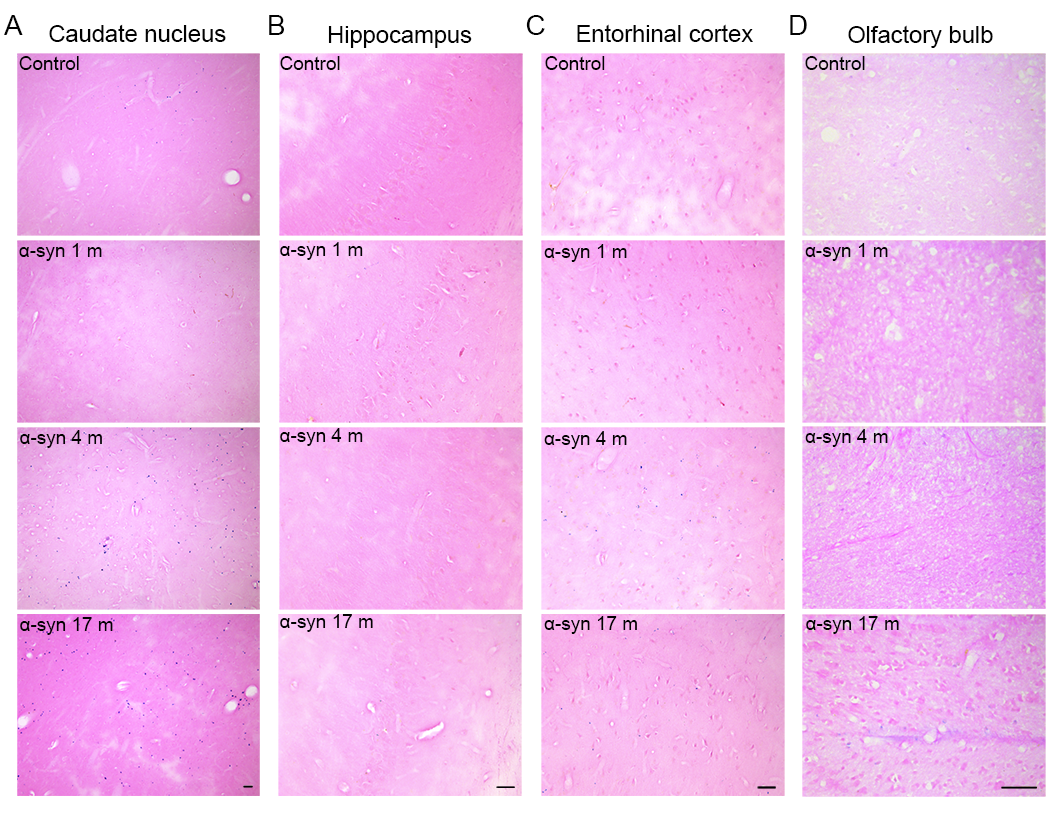

Supplement: Supplementary file 3 — Supplementary Fig. 2 [file 41419_2020_3369_MOESM3_ESM.tif]

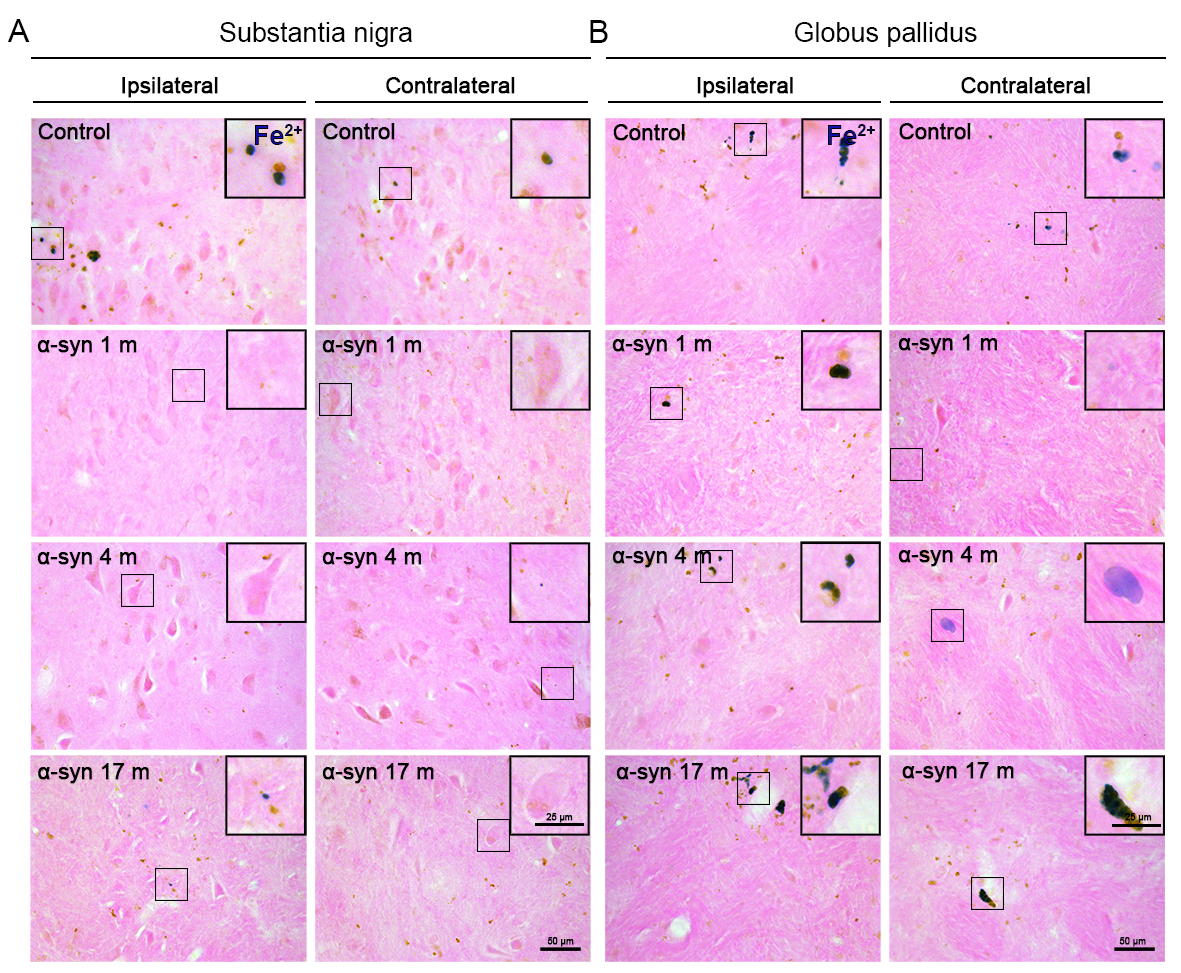

Supplement: Supplementary file 4 — Supplementary Fig. 3 [file 41419_2020_3369_MOESM4_ESM.tif]

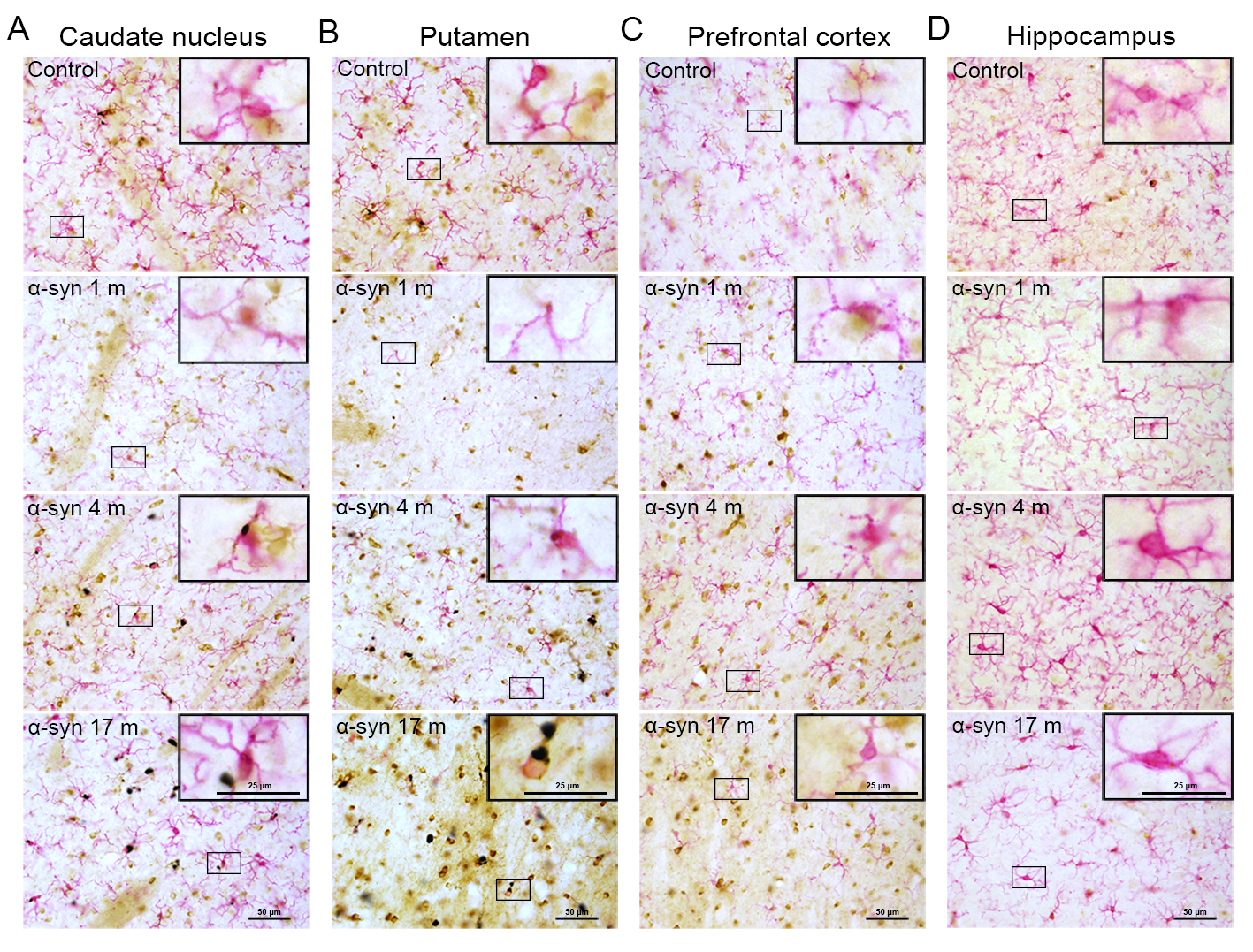

Supplement: Supplementary file 5 — Supplementary Fig. 4 [file 41419_2020_3369_MOESM5_ESM.tif]

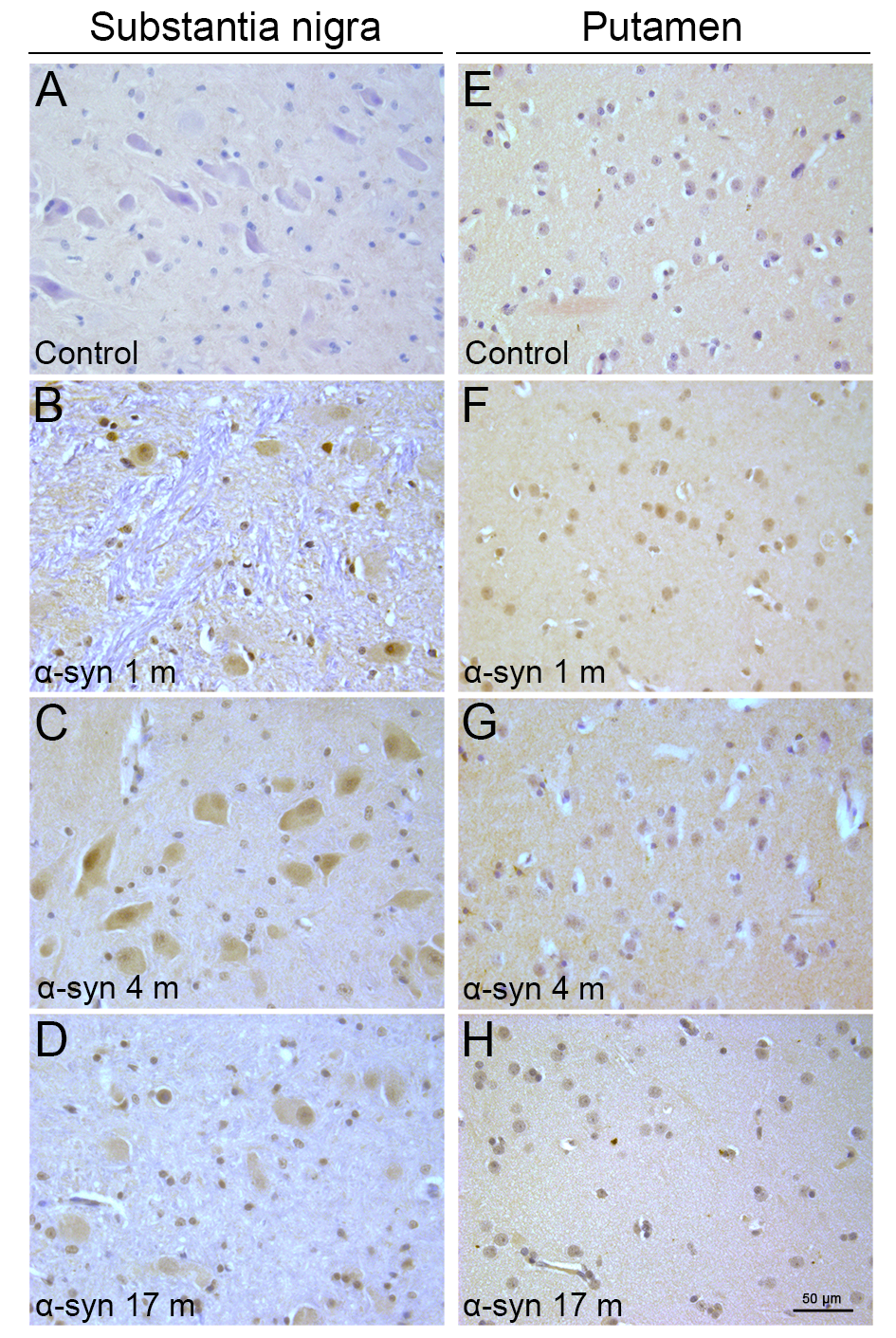

Supplement: Supplementary file 6 — Supplementary Fig. 5 [file 41419_2020_3369_MOESM6_ESM.tif]

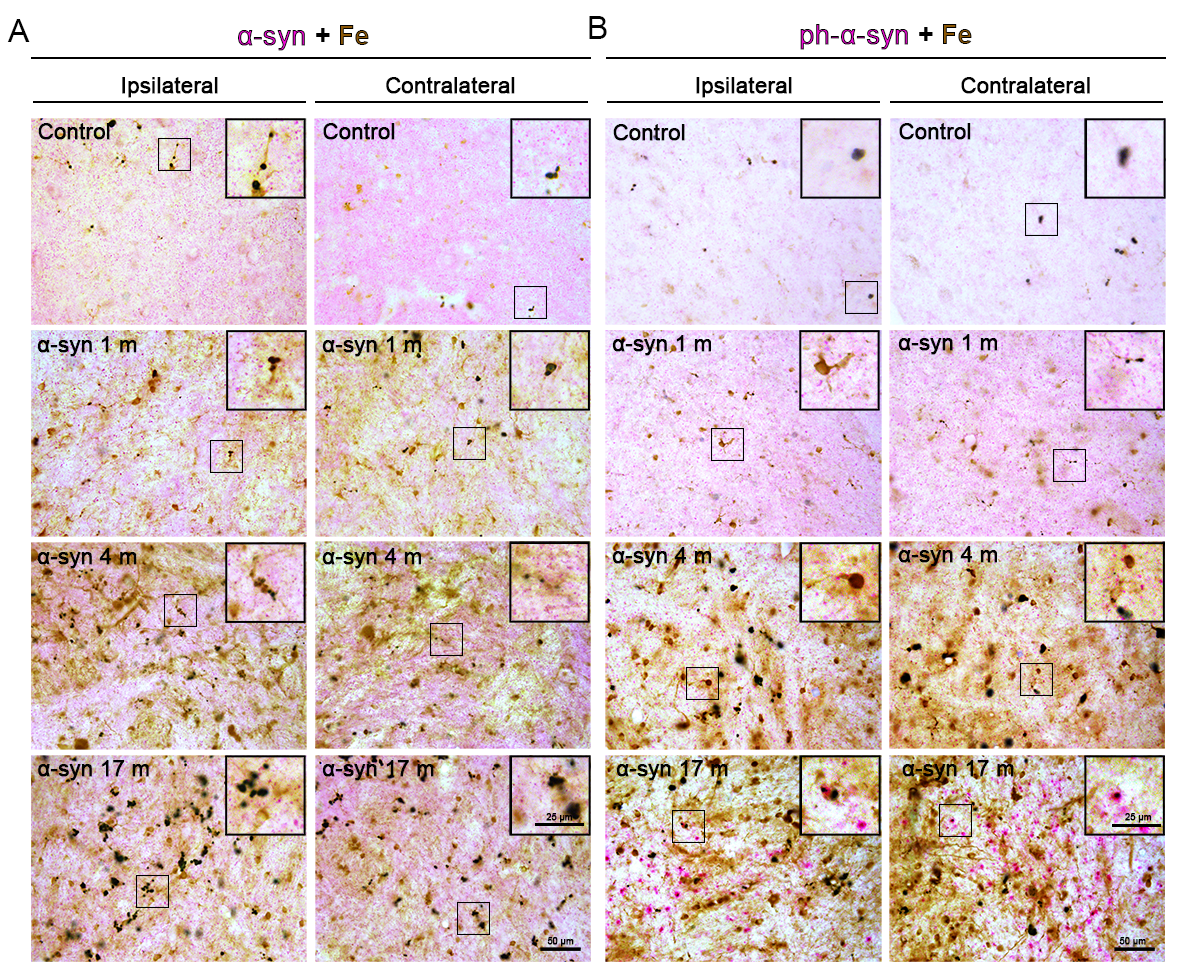

Supplement: Supplementary file 7 — Supplementary Fig. 6 [file 41419_2020_3369_MOESM7_ESM.tif]

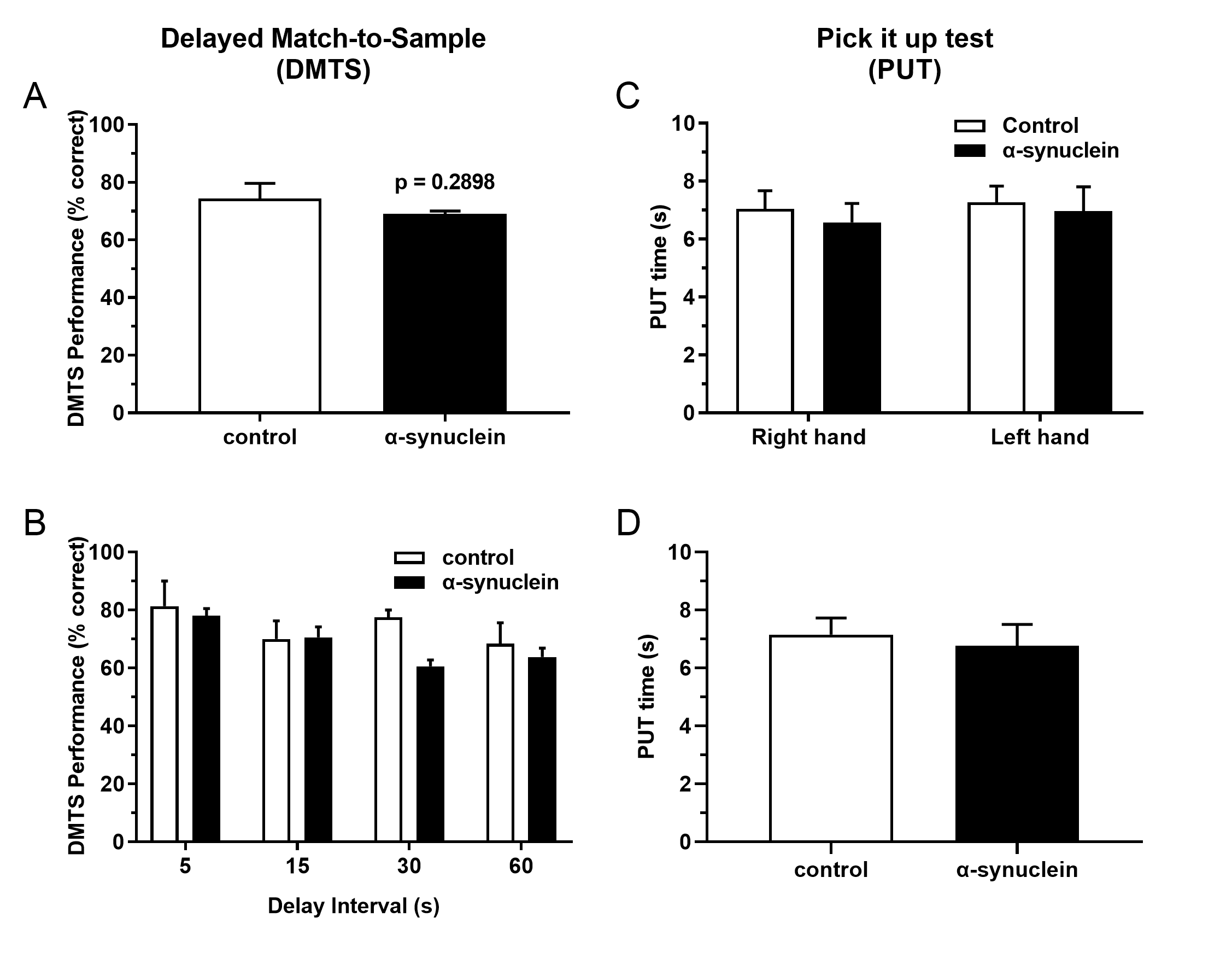

Supplement: Supplementary file 8 — Supplementary Fig. 7 [file 41419_2020_3369_MOESM8_ESM.tif]

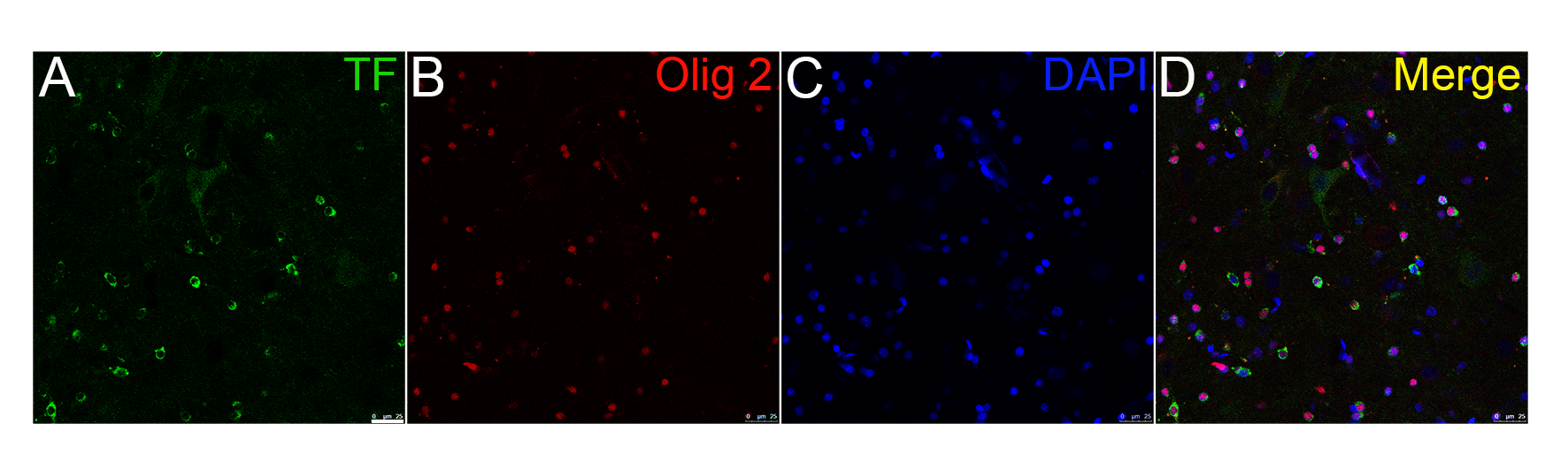

Supplement: Supplementary file 9 — Supplementary Fig. 8 [file 41419_2020_3369_MOESM9_ESM.tif]
